# Differentially Active and Conserved Neural Enhancers Define Two Forms of Adaptive Noncoding Evolution in Humans

**DOI:** 10.1093/gbe/evac108

**Published:** 2022-07-22

**Authors:** Jason Pizzollo, Trisha M Zintel, Courtney C Babbitt

**Affiliations:** Molecular and Cellular Biology Graduate Program, University of Massachusetts Amherst, Amherst, MA 01003, USA; Department of Biology, University of Massachusetts Amherst, Amherst, MA 01003, USA; Molecular and Cellular Biology Graduate Program, University of Massachusetts Amherst, Amherst, MA 01003, USA; Department of Biology, University of Massachusetts Amherst, Amherst, MA 01003, USA; Department of Biology, University of Massachusetts Amherst, Amherst, MA 01003, USA

**Keywords:** Cis-regulation, human brain evolution, induced pluripotent stem cells, massively parallel enhancer assay

## Abstract

The human and chimpanzee genomes are strikingly similar, but our neural phenotypes are very different. Many of these differences are likely driven by changes in gene expression, and some of those changes may have been adaptive during human evolution. Yet, the relative contributions of positive selection on regulatory regions or other functional regulatory changes are unclear. Where are these changes located throughout the human genome? Are functional regulatory changes near genes or are they in distal enhancer regions? In this study, we experimentally combined both human and chimpanzee *cis*-regulatory elements (CREs) that showed either (1) signs of accelerated evolution in humans or (2) that have been shown to be active in the human brain. Using a massively parallel reporter assay, we tested the ability of orthologous human and chimpanzee CREs to activate transcription in induced pluripotent stem-cell-derived neural progenitor cells and neurons. With this assay, we identified 179 CREs with differential activity between human and chimpanzee; in contrast, we found 722 CREs with signs of positive selection in humans. Selection and differentially expressed CREs strikingly differ in level of expression, size, and genomic location. We found a subset of 69 CREs in loci with genetic variants associated with neuropsychiatric diseases, which underscores the consequence of regulatory activity in these loci for proper neural development and function. By combining CREs that either experienced recent selection in humans or CREs that are functional brain enhancers, presents a novel way of studying the evolution of noncoding elements that contribute to human neural phenotypes.

**Significance**

The study examines the relative contributions of positive selected *cis*-regulatory regions versus other functional regulatory changes in the evolution of the human brain. To functionally test these contributions, we utilized high-throughput enhancer assays to measure significant differences in implicated regulatory regions, driving differential transcription between human and chimpanzee neural progenitor cells and neurons. We found that these *cis*-regulatory regions strikingly differ in level of expression, size, and genomic location. These divergent subsets of *cis*-regulatory elements may have played very different roles in human brain evolution and disease susceptibility.

## Introduction

Changes in the regulation of gene expression underlie phenotypic differences between species and individuals within a species. It was hypothesized by King and Wilson (1975) that phenotypic differences between humans and chimpanzees were due to differences in gene expression, rather than protein coding changes. Since then, changes in the regulatory elements have been shown to control differential traits between species including changes in wing pigmentation in fruit flies (Gompel et al. 2005), pelvic reduction in sticklebacks (Chan et al. 2010), and limb loss in snakes (Kvon et al. 2016). Furthermore, noncoding regulatory variants in the human population are associated with differences in disease susceptibility between individuals (ENCODE Project Consortium 2012; Schaub et al. 2012; Pai et al. 2015). Sometimes individual genetic polymorphisms are sufficient to drive phenotypes, but in many cases, traits are polygenic and multiple loci contribute to traits (Kathiresan et al. 2009; Boyle et al. 2017). In other cases, the presence of deleterious alleles alone is insufficient to manifest differential phenotypes, but rely on combination with other alleles or external factors before differential traits emerge (Cooper et al. 2013). Noncoding changes that give rise to phenotypic differences between humans and chimpanzees therefore may also contribute to differential phenotypes within the human population if other genomic variants interact. Understanding where noncoding genetic variants occur and their impacts on regulatory function are critical to understand how traits arise between species and how those changes contribute to differential phenotypes between humans.

Regulation of transcription occurs through the interaction of *cis*- and *trans*-factors that help assemble transcriptional machinery at gene promoters to active transcription (Allen and Taatjes 2015; Heinz et al. 2015). *Cis*-regulatory elements (CREs) are noncoding sequences that bind transcription factors and can activate transcription of genes, and *trans*-regulatory elements are the products of genes that regulate gene expression through interactions with DNA or other proteins. Enhancers are a class of CREs that function independent of distance or orientation to their target genes and help modulate transcription in specific ways. Elaboration of transcription profiles can change in spatiotemporal ways through the modification of enhancers of that introduce new patterns of gene expression (Rebeiz et al. 2011; Koshikawa et al. 2015). Most enhancers are located in introns of genes and intergenic regions and enhancers have been reported that are located up to 1 Mb from their target gene (Lettice et al. 2003). *Trans-*factors on the other hand are typically proteins, such as transcription factors, that bind CREs to activate transcription, but can include any diffusible molecule, such as noncoding RNA, that can influence gene expression. Although interactions between *cis-* and *trans-*factors are required for defining the transcriptional activity of a regulatory sequence, much of the specific control of transcription comes from enhancers that specify when and where genes are expressed (Long et al. 2016), which makes characterization of these elements an important part of understanding the regulation of gene expression. Many previous studies have done this in the context of model systems and detailed dissection of regulatory regions (e.g., Prabhakar et al. 2008; Capra et al. 2013; Caporale et al. 2019).

At a genomic level, both computational and experimental methods can identify putative noncoding regulatory sequences. Computational methods are often focused on identifying conserved noncoding sequences (CNSs) that have experienced minimal change through millions of years of evolution. Because of this evolutionary constraint, they are assumed to be functional, and indeed many of these have demonstrated enhancer function (Pennacchio et al. 2006; Shen et al. 2012). Experimental approaches often hinge on protein interactions with chromatin or accessibility of chromatin to infer the presence of regulatory elements. Chromatin immunoprecipitation followed by sequencing (ChIP-seq) of acetylated histones, for example, can identify putative enhancers and promoters on a genome-wide scale. Similarly, ChIP-seq for a specific transcription factor can identify CREs to which a given transcription factor binds. The physiological context in which experimental methods are used to identify putative CREs is important because chromatin accessibility can change between cell types and ChIP-seq typically identifies proteins bound to open regions of chromatin.

Both experimental and computational approaches to identify CREs have an important caveat. The elements they identify have characteristics associated with functional elements, but the level and cellular context of their activity remain unknown. The classic approach to test the function of a putative regulatory sequence is a reporter assay in which a candidate sequence is cloned into a plasmid upstream of a promoter and reporter gene and then transfected into cells to test the ability of the candidate sequence to drive expression of the reporter gene. This candidate approach uses a one-by-one testing method that is low throughput and only able to test sequences in cells amenable to transfection of the reporter construct. Leveraging next-generation sequencing, massively parallel reporter assays (MPRAs) are capable of testing thousands of candidate CREs in a single assay (e.g., Inoue and Ahituv 2015; White 2015; Girskis et al. 2021; Uebbing et al. 2021). In principle, these assays use a similar design to conventional reporter assays, but use transcribed CRE-specific unique molecular identifiers as a way of measuring reporter transcription. Given that evolution within regulatory sequences can confer adaptive phenotypes, we were interested in characterizing putative enhancers that may have had adaptive roles in shaping unique neural phenotypes in humans.

Here we used a multifaceted approach to identify putative CREs that likely have functional consequence for human phenotypes. In particular, we tested both computationally and experimentally defined CREs in both human and chimpanzee induced pluripotent stem-cell (iPSC)-derived neural cells. In contrast, other MPRA studies in human evolution have looked human accelerated regions (HARs) or histone-mark-defined CREs in human iPSC-derived neural progenitor cells (NPCs) or other neuronal cells from only humans (Ryu et al. 2018; Girskis et al. 2021; Uebbing et al. 2021). Additionally, we used a genome-integration approach, as opposed to transfection (Girskis et al. 2021; Uebbing et al. 2021). First, we chose to assay highly CNSs that have increased substitution rate in humans, including those defined as HARs (Pollard et al. 2006; Prabhakar et al. 2006; Lindblad-Toh et al. 2011; Levchenko et al. 2018). Because these sequences have higher than expected rates of substitution in humans, they may contribute to important differences in human development. They are also strongly associated with neural development and function in multiple studies (Haygood et al. 2010; Levchenko et al. 2018). Secondly, we also chose to assay sequences that have chromatin marks (H3K27ac and H3K4me2) associated with active enhancers that were identified in developing or adult human brain (Vermunt et al. 2014; Reilly et al. 2015; Vermunt et al. 2016). By choosing to assay CREs with putative roles in development or activity in neural cells, we thereby focused our analysis on CREs that may contribute to human-specific gene regulation. To learn about how human-specific changes in CREs influence transcriptional activity, we also tested orthologous chimpanzee sequences along with these candidate human CREs. This combination of CREs that experienced recent selection in humans and CREs that are functional brain enhancers presents a way of studying the evolution of both kinds of noncoding elements that contribute to human neural phenotypes.

We functionally tested candidate CREs with a lentiviral-based MPRA in iPSC-derived NPCs and neurons to assay CREs in a physiologically relevant context. Using a lentiviral vector, CRE-reporter constructs integrate into the genome, which allows for transcription from chromatin instead of an episomal vector, and allows for transduction of neural cell types that can be more sensitive to conventional transfection techniques (Inoue et al. 2017). This study is also different in that the CREs were put into species-specific iPSC-induced neurons. In this assay, we identified 179 CREs that have differential activity between human and chimpanzee orthologs and characterize sequence changes that give rise to new transcription factor–binding motifs within CREs. We also find many of the CREs in our assay in loci that contain genome wide association studies (GWAS) single nucleotide polymorphisms (SNPs) associated with neuropsychiatric diseases, which highlights the important functional consequences that can arise from variation in these noncoding regions. Finally, by looking at the genomic distribution of CREs with signs of selection in our assay, we identify and focus on the Wnt signaling pathway as a frequent target of positive selection in humans, which could have consequences for human neural phenotypes in both health and disease.

## Results

### MPRA in NPCs and Neurons Identify Differentially Expressed CREs

**Fig. 1.**
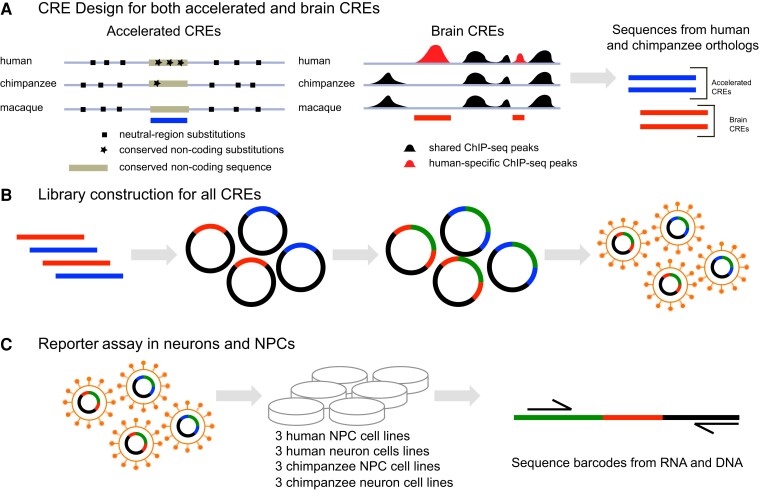
Experimental approach using MPRAs. (*A*) Accelerated CREs were identified by computational methods that (1) find conserved noncoding sequences, and (2) identify brain CREs by the presence of histone marks associated with enhancers in humans but not other primates (shown in figure), or histone marks for brain-specific enhancers. (*B*) 230-mer oligonucleotides containing CRE and barcode sequences were cloned into a lentiviral vector, a minimal promoter and EGFP reporter gene were inserted between CREs and barcodes, and plasmids were packaged in lentivirus particles. (*C*) Lentivirus-containing MPRA library was used to transduce human and chimpanzee NPCs and neurons.

To identify CREs that might have differences in function between humans and chimpanzees, we used a multipronged approach and tested genomic regions that have experienced positive selection or have active chromatin marks from brain tissue in humans (fig. 1*A*; Pollard et al. 2006; Prabhakar et al. 2006; Lindblad-Toh et al. 2011; Vermunt et al. 2014; Reilly et al. 2015; Vermunt et al. 2016). HARs are sequences of conserved noncoding DNA that have accelerated rates of substitutions in humans (Pollard et al. 2006; Prabhakar et al. 2006; Lindblad-Toh et al. 2011). Regions with histone marks associated with enhancers were (1) identified in adult or developing human brain or (2) have histone marks for enhancers in cortical areas of brain tissue human but not chimpanzee (Vermunt et al. 2014; Reilly et al. 2015; Vermunt et al. 2016).

We designed our MPRA library to test a total of 2,274 pairs of human–chimpanzee orthologous CREs. This set includes 1,579 CREs with signs of accelerated rates of substitution in human sequences, “accelerated CREs,” and 695 CREs that are associated with ChIP-seq-defined enhancers in human brain samples, “brain CREs.” All of the accelerated CREs from our literature sources were included, but the ChIP-seq data sets include many more putative CREs than we can test in our assay. To select brain CREs for our assay, we randomly chose sequences from all combined ChIP-seq sources as the other component of sequences in our library. The components of the CRE sequences were synthesized as 230 bp oligomers (fig. 1*A*) that were cloned into a lentiviral transfer plasmid. The final lentiviral reporter constructs have CRE sequences upstream of a minimal promoter and EGFP reporter gene, each with a unique barcode in the 3′-UTR. The lentiviral MPRA library was packaged into viral particles and used to transduce iPSC-derived NPCs and neurons (fig. 1*B and C*). We tested three biological replicates from different individuals for each human and chimpanzee and for both iPSC-derived NPCs and neurons, with a total of 12 assays. Total RNA and DNA were collected 48 h after infection and barcode amplicons were sequenced to quantify CRE expression. The counts represent a sum of the reads across CREs.

**Fig. 2.**
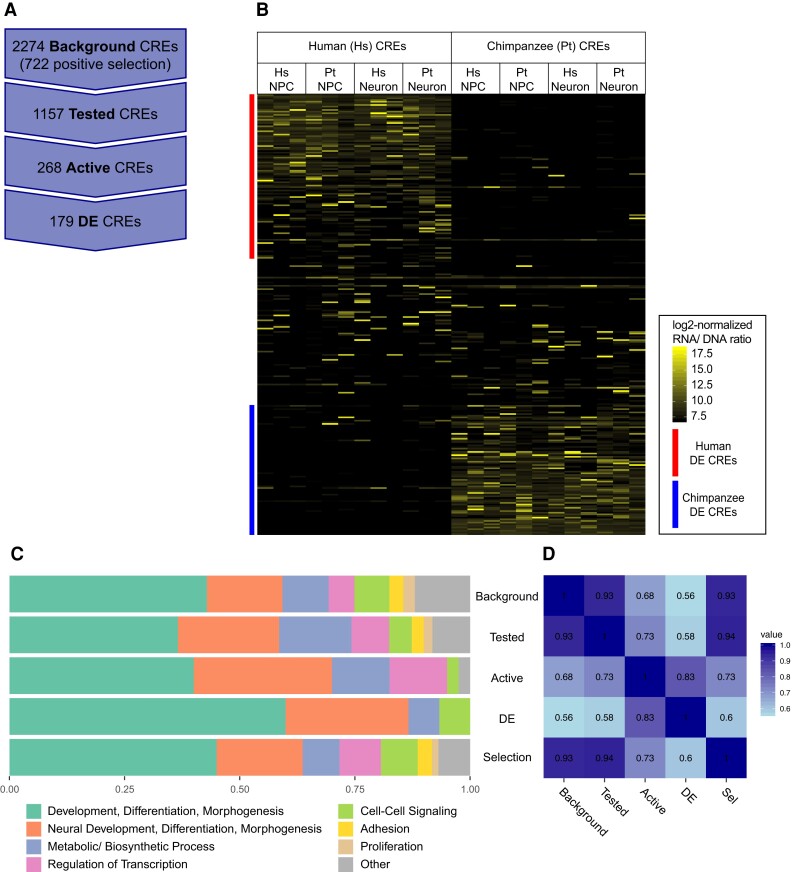
CREs exhibit distinct subsets of expression and function. (*A*) Background CREs include all 2,274 orthologous CREs in designed array. Selection tests identified 722 with signs of selection in human sequences. (*B*) Heatmap of log_2_-normalized RNA/DNA ratio for human CREs and chimpanzee CREs (268 orthologs), vertical bars on the left side of the plot indicate CREs that show significantly different levels of expression between orthologous CREs. (*C*) Proportion of enriched GO biological process terms in broad category types for each set of background, tested, active, DE, and selection (Sel) CREs. (*D*) Semantic similarity of enriched GO terms between CRE subsets.

Our initial library consisted of 2,274 CREs in the designed array. As not all of these were active in our assay, we refer to these as the “background” set in subsequent analyses as they are a subsampling of regions found in the human and chimpanzee genomes (see Methods). We found 1,157 CREs that were expressed by both species orthologs in these cells, and further filtered these to 268 that were active in at least 2 of 3 replicates of either species cell type (fig. 2*A*). Other CREs in the assay were not necessarily “inactive,” and they simply were not represented in enough cell types or replicates for us to robustly compare expression across species (there were 643 CREs with only one replicate expressed). We used limma (Ritchie et al. 2015) to test for differential expression between orthologous CREs across all replicates and found 179 CREs that have significantly different (*P* ≤ 0.05) levels of activity between orthologs (fig. 2*B*).

### Investigating CREs for Signs of Positive Selection

To investigate adaptive noncoding changes, we included a subset of CREs in our assay had signs of accelerated evolution in humans. As the CREs in our assay came from different sources and a subset of those had not been tested for signs of selection (from chromatin assays, not bioinformatic scans), we performed our own test for positive selection in the human putative CREs. We used the method described by (Haygood et al. 2007) and executable in HyPhy (Pond et al. 2005). This test calculates nucleotide substitution rates in a given noncoding sequence, using sequence alignments across human, chimpanzee, and macaque (used as an outgroup), and compares that to a neutral substitution rate approximated from intronic (excluding first introns) and intergenic regions within 100 kb of CRE sequences. It is analogous to a d*N*/d*S* test, where a higher substitution rate in the CRE sequence compared with the neutral sequence is used to infer positive selection. Not all sequences aligned between the human, chimpanzee, and macaque genomes, so we bioinformatically tested 1,891 putative CREs for which sequence alignments were available for all three species. We found 722 with signs of positive selection using a likelihood ratio test with a significance *P*-value ≤0.05. We classified selection in CREs based on individual *P*-values instead of adjusting for multiple testing to make the selection criteria as inclusive as possible. We defined this set of “selection CREs” as a way to look at the activity and characteristics of enhancers that experienced recent evolution in humans.

### Gene Ontology Enrichments are Similar Across CRE Sets

We next wanted to see if CREs in different subsets represent similar biological processes; hence, we compared enriched gene ontology (GO) terms between selection, background, tested, active, and differentially expressed (DE) CREs. While the tested and active CREs represent portions of the background set after filtering for activity in our assay, selection and DE CREs belong to fairly distinct groups. The selection CREs have signs of accelerated substitution in humans, whereas the DE CREs have significant differences in activity between human and chimpanzee orthologs. Between 722 selection and 179 DE CREs, there are only 34 CREs that have signs of selection and are DE between orthologs, demonstrating that the selection and DE CREs are mostly distinct sets. We found that brain CREs are more likely to be active and DE when compared with selection CREs (Fisher’s exact test, *P* = 3.909e-05). This is possibly due to the selection CREs being utilized in different cell types or developmental stages that we were not able to assay here.

We assigned CREs to their nearest 5′ and 3′ genes with GREAT (McLean et al. 2010) and performed GO enrichment analysis with gProfiler (Raudvere et al. 2019). For the 34 CREs that have signs of positive selection and DE, we only found significant enrichment (*q* < 0.05) of two KEGG pathways: carbohydrate digestion and absorption, and cysteine and methionine metabolism (supplementary table S1, Supplementary Material online). For other CRE sets, we grouped enriched terms (*q* ≤ 0.05) into broad category types to see if different types of processes were similarly represented in CRE sets, and tested similarity of enriched terms between CRE sets using GO SemSim (Yu et al. 2010) which compares semantic similarity between sets of GO terms and is a way to quantify similarity between sets of enrichments.

Although the sets of tested and selection CREs have similar representation of different types of biological processes and a high measure of semantic similarity to background, the active and DE CREs look to have less diversity in their representation of biological processes and lower semantic similarity scores (fig. 2*C and D*). In order to investigate if this was because these sets have fewer CREs or if the CREs in these sets represent a narrower range of processes, we drew 10 random samples of 179 CREs (the same as the number of DE CREs) from the background set and compared the enriched GO terms. The randomly sampled sets often (6/10) show more diversity of enriched terms and more similar semantic similarity scores than DE CREs (supplementary fig. S1, Supplementary Material online). There are some (4/10) randomly sampled sets, however, that have similar or lower diversity than DE CREs, which suggests the decreased diversity of enriched terms from active and DE CREs is due to the smaller number of CRE in these sets.

### Selection CREs are More Distal to Genes and Other Enhancers

Putative CREs occur widely throughout the genome but their genomic locations are not random. The distances between CNSs, and between CNSs and the nearest gene, are conserved between avian and mammalian genomes (Sun et al. 2006; Babarinde and Saitou 2016) and there is evidence that pairs of adjacent CNSs act together in a distance-sensitive way (Li et al. 2018). Genomic locations may also inform about function of regulatory elements. Zabidi et al. (2015) report that housekeeping enhancers overlap or are proximal with transcription start sites (TSSs), whereas developmental enhancers are mostly intergenic or intronic.

**Fig. 3.**
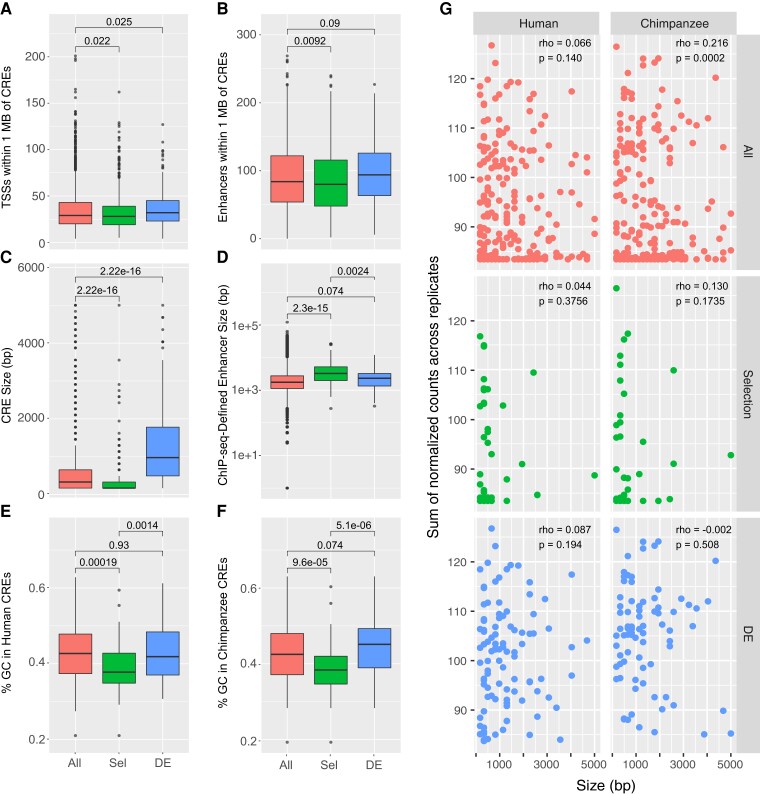
Different genomic characteristics of selection and DE CREs. (*A*) The number of TSSs located within 1 Mb of background, selection (Sel) and DE CREs. Boxplots show median (horizontal black bar), first and third quartiles (upper and lower limits of boxes), whiskers (1.5 times the IQR, the third minus first quartile), and individual points (values beyond 1.5*IQR). (*B*) Number of active brain enhances within 1 Mb of CREs. (*C*) Size distribution of CREs. (*D*) Size distribution of active brain enhancers that contain CREs. (*E*) GC content in human and (*F*) chimpanzee CREs. (*G*) Plot of activity against size for human and chimpanzee CREs. Activity is the sum of log_2_-normalized RNA/DNA counts across all 12 replicates. Spearman correlation tests show relationships between activity and size.

Given that there are differences in the genomic distribution of CREs, we were interested to learn if there are particular genomic characteristics that are more associated with CREs under selection or CREs that are DE between orthologs. We first wanted to look at where CREs are located in relation to nearby genes and other putative regulatory elements. To look at genomic locations, we downloaded reference data sets for TSSs from Ensembl release 98 (Zerbino et al. 2018), and used histone acetylation and methylation ChIP-seq data sets from our brain CRE sources (Vermunt et al. 2014; Reilly et al. 2015) to identify other neural-active regulatory elements. For each CRE, we counted the number of annotated TSSs and putative enhancers within 1 Mb. Selection CREs are generally near fewer TSSs and putative enhancers than the background CRE set, whereas DE CREs are in regions with greater numbers of TSSs but not necessarily more enhancers (fig. 3*A and B*). Although the average differences are not great, they are statistically significant differences in trend in genomic location between selection CREs and DE CREs.

### Selection CREs are Smaller but Belong to Larger Regulatory Domains

We were next interested to see how the size of regulatory elements differs between CRE sets. Based on CRE origins and tissue specificity, there is some evidence that different types of CREs differ in size. For example, Moon et al. (2019) show that there are no significant differences in length between enhancers with signs of positive selection and those without selection, but there are differences in enhancer length specifically in brain and testis enhancers. We looked at the size distribution of CREs in our assay and found that CREs with signs of selection are significantly smaller than background, whereas DE CREs are significantly larger than background (fig. 3*C*). However, the size difference between CREs is influenced by their original identification methods. Most selection CREs were identified computationally by searching for highly conserved sequences in animals. HARs have an average size of 257 bp and more evolutionarily ancient mammalian CNSs are even shorter (Capra et al. 2013). In our assay, selection CREs, most of which are HARs, are contrasted with CREs identified from ChIP-seq data sets that have an average size of 1,932 bp, so part of the difference in size that we see is due to the methods that were originally used to define these CREs.

To further compare selection and DE CREs, we looked for overlap between our CREs and functionally active regulatory elements in the human brain (Vermunt et al. 2014; Reilly et al. 2015). We compared the size of the brain enhancers that overlap our selection or DE CREs and found that selection CREs overlap with larger regions compared with DE CREs (fig. 3*D*). The larger regions to which selection CREs belong may then be related to the way that enhancers evolved around regions with highly conserved sequences. Sequences adjacent to highly conserved regions can evolve functions to help modulate ancestral expression patterns in new spatiotemporal ways. Over time, addition of new functional elements can increase the size of an enhancer (Emera et al. 2016).

Larger CREs could contain more functional elements that contribute more transcriptional output and thus higher activity. Indeed, DE CREs are larger and have higher activity than background and selection CREs (fig. 3*G*). We wanted to determine if increased activity is due to larger CRE size, so we tested for a correlation between size and activity. Overall, we did not find a strong relationship and only see a weak correlation in chimpanzee CREs (Spearman’s rho = 0.216, *P* = 0.0002). Hence, although DE CREs are larger, it does not seem that their higher activity is due to their larger size.

### Selection CREs have Significantly Lower GC Content

One of the sequence characteristics that can differentiate enhancers is GC content. Chen et al. (2018) report a positive correlation between enhancer GC content and activity, and Colbran et al. (2017) report that GC dinucleotides are associated with broad enhancer activity. Overall, the GC content in the human genome is about 42%, but that rate can be lower in CNSs. In sequences that are conserved among mammals, the GC content is lower than background ∼37%, whereas those that are only conserved among primates have GC content that is similar to the genomic background (Babarinde and Saitou 2013). This difference is likely due to the presence of lineage-specific TF motifs that have lower GC content compared with ubiquitous TF motifs (Hettiarachchi and Saitou 2016). Changes in GC content can also occur in the genome by the recombination driven process of GC-biased gene conversion (gBGC) (Galtier and Duret 2007). Although this is a neutral process, changes in GC content in CNSs could contribute to an increased substitution rate that mimics positive selection (Kostka et al. 2012).

We looked at the GC content of CREs in our assay and found that selection CREs have lower GC content than background in both human and chimpanzee sequences, whereas GC content in DE CREs is not significantly different from background (fig. 3*E and F*). The lower GC content that we observed in selection CREs suggests that gBGC is not driving selection signals in these CREs. The proportions of G and C nucleotides in selection CREs are in agreement with the reported decreased GC content in mammalian conserved CNSs and supports putative functions for selection CREs as lineage-specific developmental enhancers. This contrasts with DE CREs that may have arisen more recently and perhaps function in a more general way. We also looked for correlations between GC content and CRE activity within each CRE set, but overall we do not see a strong relationship. There are weak positive correlations in chimpanzee CREs (Spearman’s rho = 0.114, *P* = 0.031) and human DE CREs (Spearman’s rho = 0.179, *P* = 0.038), but these values are low and do not indicate that GC content is driving differences in activity (supplementary fig. S2, Supplementary Material online).

We were next interested to see if CpG content differed between CRE sets. CpG deamination was previously identified as a significant mechanism of sequence evolution in primate enhancers (Klein et al. 2018). We scanned all of our CREs for CpG motifs and found lower CpG content in chimpanzee selection CREs compared with background (supplementary fig. S3, Supplementary Material online). It does not seem that this difference is because of lower GC content; however, human CREs also have low GC content but not significantly lower CpG motifs. A slightly but not significantly higher number of CpGs was reported in human than chimpanzee. These data suggest that CpG deamination is not a strong driver of sequence evolution in the CREs that we assayed.

### Selection CREs have Fewer TF Motifs but More TF Gains in Humans

Enhancer activity is largely defined by the binding of transcription factors to specific motifs, which results in the recruitment of transcriptional machinery to promoters to activate transcription of a target gene. As TF motifs are the basic functional units within enhancers, we wanted to see if there were differences in motif abundance between CREs in our assay. To explore this, we scanned human and chimpanzee CRE sequences for JASPAR motifs (Khan et al. 2018) using FIMO (Grant et al. 2011) with a *P*-value cutoff of 10^−4^. We first looked at the overall density of TF motifs in selection, DE, and background CREs and compared that to random noncoding genomic regions (see Materials and Methods) to see if TF motif density was higher in our CREs than the genomic average. To normalize for CRE size, we calculated TF motif occurrences per 100 bp. Across all sequences tested, the average motif density is ∼15 motifs/100 bp, but in both human and chimpanzee CREs, the average is slightly higher than genomic background, with the exception of human selection CREs that are slightly lower (14.9 motifs/100 bp; supplementary fig. S4A, Supplementary Material online). Although the distributions overlap across all sequences tested, the average motif density in both human and chimpanzee selection CREs is significantly (*P* < 0.006) lower in all comparisons (supplementary fig. S4B, Supplementary Material online).

**Fig. 4.**
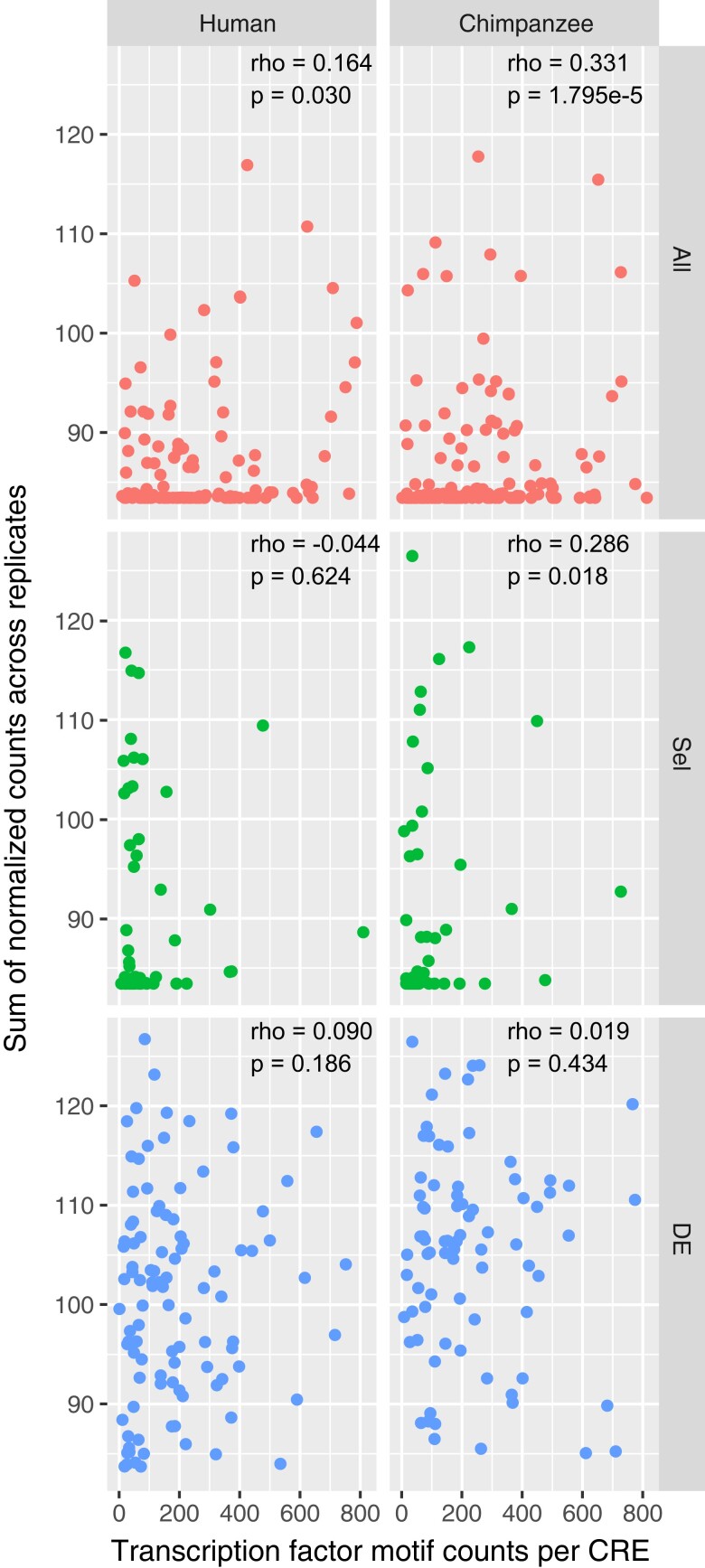
Activity versus transcription factor motif occurrences. The sum of log_2_-normalized RNA/DNA counts across all replicates is plotted against the total number of transcription factor motif occurrences in each CRE. Spearman correlation testes show relationships between motif occurrences and CRE activity.

In general, the activity of selection CREs is lower in our assay compared with DE or background CREs (fig. 3*G*), so we checked if there was a relationship between TF motif density and CRE activity by comparing the normalized counts across all replicates for each CRE to the total number of TF motifs per CRE. For all human and chimpanzee background CREs, there is a weak-to-moderate positive correlation between motif number and activity (Spearman’s rho = 0.164, *P* = 0.030 human; rho = 0.331, *P* = 1.795e-5 chimpanzee; fig. 4). There is also a similar relationship in chimpanzee selection CREs (rho = 0.286, *P* = 0.018), but not in other CRE sets. Overall, the abundance of TF motifs in a sequence is not a strong predictor, but in some cases may inform about CRE activity. The presence of a TF motif in a DNA sequence does not necessarily indicate that a given TF binds at that location. Likewise, the absence of a TF motif does not mean that a TF does not bind a particular sequence. TF binding can occur in a cooperative way such that nearby transcription factors bound to an enhancer can recruit cofactors to sequences that do not match predicted motifs (Rubinstein and de Souza 2013; Grossman et al. 2017). TF binding to enhancers is context dependent, but nonetheless, scanning for motifs can provide insight into which sites in an enhancer might be active and how sequences are evolving.

As changes in TF motif abundance can contribute to differential activity, we next looked at global differences in TF motif occurrences between human and chimpanzee CREs. For each motif, we calculated the total number of occurrences in human and chimpanzee sequences. To find motifs that occur at significantly different frequencies between species, we focused on those for which the fold change between human and chimpanzee was at least 2 standard deviations from the mean, and performed this calculation for all background, selection, and DE CREs.

Across all CREs, there are 17 motifs that are significantly more abundant in humans and 4 that are more abundant in chimpanzee (supplementary table S2, Supplementary Material online). In human CREs, *MSX3*, *MSX2*, *CUX2*, and *CEBPG* occur at 15–35% increased frequency compared with chimpanzee and these are transcription factors that are expressed in the brain. In chimpanzee CREs, *ZNF740*, *GMEB1*, and *PDX1* motifs are 14–19% more abundant than in human, whereas *LBX1* motifs are 113% more abundant.

We wanted to know if there were particular motifs that experienced significant changes in frequency in both the selection and DE CREs, but also wanted to separate out background effects due to global changes in TF frequency between species. To do this, we calculated the difference in the human–chimpanzee fold change between selection or DE CREs and background. We then focused on motifs that were changing significantly more or less (≥2 standard deviations from the mean fold-change difference) in selection and DE CREs. This shows us the motifs that are changing at a much faster rate in selection and DE CREs compared with background changes. In both human and chimpanzee DE CREs, the numbers of motifs with increased and decreased rate of change compared with background are similar (supplementary table S2, Supplementary Material online). This could suggest that these regions are experiencing a higher rate of TF turnover and could be regulatory sites that are under less constraint. In human DE CREs, there are three motifs, *CUX2*, *CBP*, and *ZBTB33*, that changing ∼50% higher human-gain rate than background. In chimpanzee DE CREs, CREM and GMEB2 experienced a much faster loss than background. In the selection CREs, there are 12 motifs that are changing significantly more between species than the background including *GMEB1/2*, *ZBTB33*, *CEBPD/G/E/B*, and *CREB3*, and only 1 motif (E3F3) that is significantly more depleted in human selection CREs than background (supplementary table S2, Supplementary Material online). This suggests that positive selection in human CREs favors the appearance of specific motifs more than the depletion of motifs, and that these changes are not due to general trends toward increased or decreased frequency of motifs in the human genome.

### Neuropsychiatric SNPs are Enriched in Loci with DE CREs

CREs that contribute to differential gene regulation between humans and chimpanzees may contribute to human-specific neural phenotypes. At the same time, regulatory changes that bring about adaptive physiological changes could contribute to increased human susceptibility to neuropsychiatric diseases (Won et al. 2019). GWAS SNPs associated with neuropsychiatric diseases fall mostly in noncoding regions of the genome (Schaub et al. 2012; Gallagher and Chen-Plotkin 2018), which supports the hypothesis that regulatory variation is an important driver of disease phenotypes. As a way to explore how regulatory changes could be related to physiological processes important for human-specific neural phenotypes, we looked for loci that contained both CREs active in our assay and neuropsychiatric SNPs. We first collected GWAS SNPs from the NHGRI-EBI catalog (Buniello et al. 2019) associated with Alzheimer’s disease, autism, attention deficit hyperactivity disorder, bipolar disorder, major depressive disorder, or schizophrenia. We then associated SNPs and putative CREs with their nearest genes using GREAT. With this approach, we found 85 genes associated with 69 CREs, 46 of which are DE CREs, and 85 SNPs (supplementary table S2, Supplementary Material online). Interestingly, we found that SNPs are much more frequently associated with DE CREs than with background CREs in our assay (Fisher’s exact test *P* = 3.4e-4), suggesting variation in loci that have unique patterns of activity in humans can also contribute to disease risk. Genes associated with CREs and SNPs represent a large range of biological processes that have important roles in development and neural function including axon growth and guidance, synapse formation, cell adhesion, DNA replication, calcium signaling, glutamate signaling, Wnt signaling, and regulation of transcription.

**Fig. 5.**
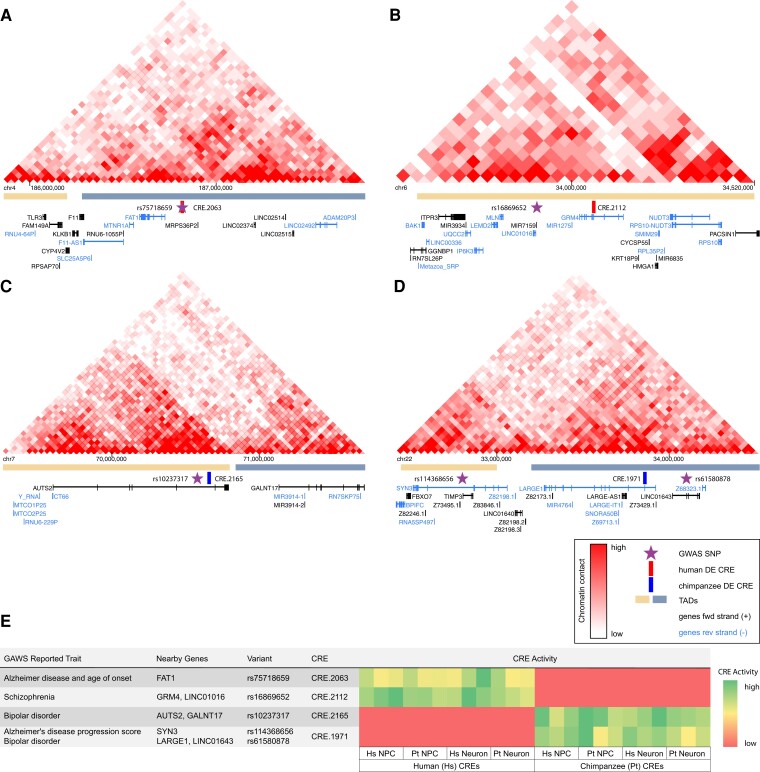
Overlaying CREs, chromatin contact maps, and neurological disease GWAS loci. (*A–D*) Hi-C data generated from prefrontal cortex by Schmitt et al. (2016) showing chromatin contacts in loci containing GWAS SNPs and DE CREs at the marked locations. Chromatin contacts are shown at 40 kb resolution (each individual square is 40 kb). Gene annotations under each Hi-C map were part of the 3D genome browser Wang et al. (2018) visualization but do not include unannotated transcripts. (*E*) Table describing GWAS SNPs, traits, nearby genes, and CREs in each locus. Log_2_-normalized RNA/DNA counts are shown for orthologous CREs in each of 12 replicates.

We define GWAS loci as the regions around genes that share at least one SNP and one CRE. Spatially, overall, we do not see clear patterns in the locations of SNPs and CREs relative to genes. The median size of the loci we identified is 327 kb. About 70% of CREs and ∼72% of SNPs occur in introns, and ∼23% of CREs and ∼22% of SNPs are intergenic. The average distance between SNPs and the closest TSS is ∼300 kb and, on average, CREs are about 335 kb away from TSSs. Given that noncoding SNPs contribute to diseases by impacting regulation of gene expression, this highlights the fact that variation in noncoding regions, even those that are quite far from target genes, can have impacts on phenotypes. While CREs putatively function through regulation of target gene transcription, SNPs in noncoding regions could influence gene expression through modification of distal enhancer function or could be part of unannotated transcripts or RNA genes, that act in *trans* to modulate expression of target genes. Although we do not have data that describe chromatin interaction in our specific cell lines, Hi-C data sets that identify genome-wide chromatin interactions (van Berkum et al. 2010) in comparable cell types or from human brain regions can shed light on how distal noncoding regions can interact with surrounding genes (fig. 5). Understanding the contributions of individual CREs on gene expression is difficult because many regulatory elements can help control the expression of a target gene. Using global RNA-seq data, we looked for correlations between gene expression and CRE activity for CRE-gene assignments made using GREAT, but did not find a correlation between the activity of regulatory elements and their closest genes (Spearman’s rho = 0.01, *P* = 0.4417). Using other approaches by layering chromatin contact information and generating additional enhancer activity measurements at more sites around loci of interest could improve our understanding of how individual enhancers contribute to regulation of gene expression, but assessment of enhancers at that regulation is beyond this scope of this study.

To look more closely at how regulatory changes could be impacting gene expression in the human brain, we looked for loci that have GWAS SNPs and CREs that are DE between species. We also used Hi-C data from human prefrontal cortex (Schmitt et al. 2016) to visualize how SNPs and CREs might be interacting with surrounding genomic regions. These data inform about long-range interactions that form topologically associated domains (TADs) through DNA looping and bring distal parts of chromosomes into close physical proximity (Dixon et al. 2012). Although the CRE and SNP locations relative to putative targets vary between all CRE–SNP associations, we focus on several loci to highlight these relationships (fig. 5). The activity of the following CREs was such that they were active in all cell types, in all replicates, and are active in other studies (Emera et al. 2016).

In the region around FAT1, an atypical cadherin, CRE.2063, and rs75718659, an Alzheimer’s disease–associated variant (fig. 5*E*), are located 1.2 kb away from each other and ∼98 kb upstream of FAT1 (fig. 5*A*). Chromatin interactions in this region suggest that CRE.2063 and rs75718659 could physically interact with the FAT1 promoter and with several upstream long noncoding RNA genes. In several cases, we see CREs located in the introns of genes. In AUTS2, which is part of a chromatin remodeling complex and is linked to autism spectrum disorder, CRE.2165 and rs10237317, a variant associated with bipolar disorder (fig. 5*E*), are located in the fifth intron (fig. 5*C*). The 3′-end of AUTS2 aligns with the boundary of two TADs, which suggests that contacts between AUTS2 and downstream sequences are limited. In another region, CRE.1971 is in the second intron of LARGE1, a glycotransferase that is required for α-dystroglycan binding to laminin and neurexins (Inamori et al. 2016; fig. 5*D*). Downstream of LARGE1, but in a separate TAD, is SYN3, a synapsin gene associated with synaptic vesicles that has rs114368656, a variant linked to Alzheimer’s disease and bipolar disorder (fig. 5*E*) in the sixth intron. In another locus, CRE.2112 is located in the third intron of GRM4, a metabotropic glutamate receptor. These features are located inside their putative targets and have some evidence of interactions with their respective promoters and neighboring regions. Chromatin interaction data suggest that frequency of interactions increase with proximity to TSS and that TSS interactions with upstream elements are more common (Sanyal et al. 2012), so their putative functions of proximal and upstream elements are more easily defined. In loci where variants are located in intergenic regions, long noncoding RNAs, or unannotated transcripts, target genes, and their role in neural phenotypes are less clear. Variant rs61580878, associated with bipolar disorder (fig. 5*E*), is located in the third intron of LINC01643, a long noncoding RNA (fig. 5*D*), and variant rs16869652, associated with schizophrenia (fig. 5*E*), located in an intergenic region ∼80 kb upstream of the motilin gene MLN. This gene shows expression in the brain, but its annotated function as a regulator of gastrointestinal contractions makes its consequence for neural phenotypes less clear. Layering these putative enhancers with other -omics data sets increased our power to find regulatroy regions with possible disease-related phenotypic effects.

### Many Genes in the Wnt Pathway have Signs of Positive Selection in Surrounding Noncoding Regions

Evolution within regulatory sequences can act in subtle ways to fine-tune CRE activity (Farley et al. 2015). In many cases, it may not be beneficial to make significant changes in a regulatory network. Highly conserved developmental pathways, for example, may be sensitive to regulatory changes that could have broad effects on development. However, as we explored CREs associated with neural development and function, we found an abundance of CREs around genes associated with canonical Wnt signaling. Throughout the pathway, we found CREs with signs of positive selection and in general, DE CREs associated with genes that modulate Wnt signaling. Additionally, when we looked at GWAS SNPs near CREs in our assay, we were surprised to see DE and selection CREs around genes in the Wnt pathway, which is involved in early developmental pattering, cell differentiation, and proliferation, and has important roles in embryonic and adult neural development and function (Patapoutian and Reichardt 2000; Logan and Nusse 2004; Ciani and Salinas 2005). Considering the Wnt pathway has essential roles in early development and neural function, we focused on Wnt to explore how selection could be acting to influence a core developmental pathway.

To look at how selection might be acting around genes involved in Wnt signaling, we looked for CREs with signs of positive selection within 500 kb of genes in this pathway. We collected genes that are part of annotated gene sets in the mSigDB (Subramanian et al. 2005) under the headings “Wnt signaling,” “GO canonical Wnt signaling pathway,” and “KEGG Wnt signaling pathway.” We then looked to see if any of the 722 CREs with signals of positive selection in our assay were within 500 kb of canonical Wnt pathway genes or if DE CREs were associated with these genes. Some genes that were part of annotated Wnt pathway gene sets do not have specific functions that are well defined, so we focused our analysis on genes that have well-defined functions in this pathway. In total, we found 57 CREs within 500 kb of 46 genes in the Wnt pathway (supplementary table S4, Supplementary Material online). Of these, 42 are selection CREs and 16 are DE CREs, whereas only one is both DE and under selection.

**Fig. 6.**
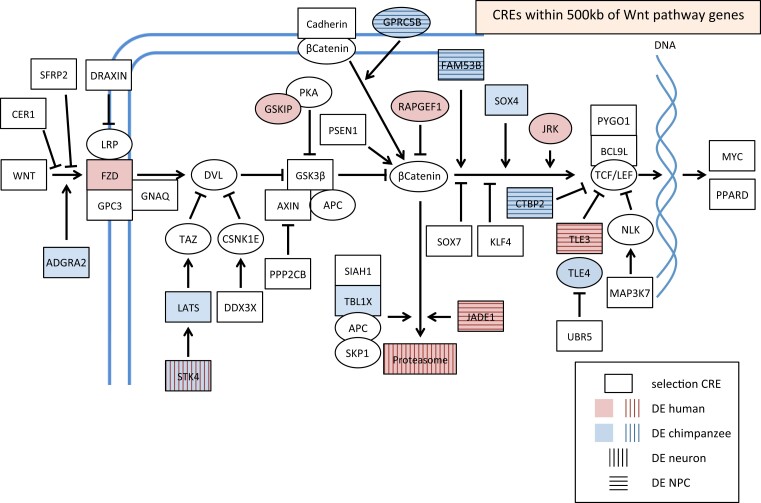
Genes modulating canonical Wnt signaling associated with selection and DE CREs. CREs were associated with genes if they occur within 500 kb of TSSs. Genes associated with selection CREs have square outlines. Genes associated with CREs that are DE between orthologs have red (higher in human) or blue (higher in chimpanzee) fill. Genes associated with CREs that are DE between cell types are marked with horizontal (higher in NPC) or vertical (higher in neuron) stripes, and stripe colors indicate if the human (stripes) or chimpanzee (stripes) CRE was DE. The selection and DE profiles of CREs associated with paralogous genes are combined for each gene in the pathway but are separately defined in Table 1. Genes with circular outlines and white color are not associated with CREs in our assay but are included to inform about relevant interactions.

CREs with signs of positive selection are associated with many genes in the canonical Wnt signaling pathway (Komiya and Habas 2008; MacDonald et al. 2009), and also with many of the genes that modulate Wnt signaling (see Materials and Methods for references; fig. 6). Some of these genes are associated with multiple CREs that have different selection and expression profiles. These data were condensed in our representation but are elaborated in table 1. Some of the major genes in this pathway have CREs with signs of selection including *WNT2*, *WNT5A*, and *WNT8B*, and Frizzled receptors *FZD5*, *FZD6*, and *FZD7*. Additionally, the human ortholog of a CRE near *FZD1* is DE. Frizzled proteins are the primary receptors for Wnt ligands, and differential expression and function of a human accelerated enhancer was previously described near *FZD8* (Boyd et al. 2015). The primary mediator of Wnt signaling is *β-catenin* that helps regulate transcription of Wnt target genes. Levels of *β-catenin* in the cell are controlled in part by the kinase *GSK3β* that phosphorylates and leads to degradation of *β-catenin*. Thus, *GSK3β* is one of the core proteins in this pathway and has an enhancer under positive selection ∼170 kb away.

**Table 1.** Wnt Genes Associated with Multiple CRE Expression or Selection Profiles

Gene	CRE	CREs Under Positive Selection	Differential Expression between Orthologs	Differential Expression between Cell Types
ADGRA2	CRE.2202	No	Chimpanzee higher	No
ADGRA2	CRE.1384	Sel	No	No
AXIN2	CRE.560	Sel	No	No
AXIN2	CRE.561	Sel	No	No
CDH2	CRE.575	Sel	No	No
CDH3	CRE.502	Sel	No	No
CDH3	CRE.503	Sel	No	No
CER1	CRE.1463	Sel	No	No
CER1	CRE.1465	Sel	No	No
CSNK2A1	CRE.799	Sel	No	No
CSNK2A1	CRE.800	Sel	No	No
CTBP2	CRE.1678	No	Chimpanzee higher	Chimpanzee CRE higher in NPC
CTBP2	CRE.136	Sel	No	No
FAM53B	CRE.1678	No	Chimpanzee higher	Chimpanzee CRE higher in NPC
FAM53B	CRE.136	Sel	No	No
FZD1	CRE.2170	No	Human higher	No
FZD5	CRE.776	Sel	No	No
FZD6	CRE.1413	Sel	No	No
FZD7	CRE.765	Sel	No	No
GNAQ	CRE.1500	Sel	No	No
GNAQ	CRE.1501	Sel	no	No
JADE1	CRE.2048	No	Human higher	Human CRE higher in NPC
JADE1	CRE.1057	Sel	No	No
JADE1	CRE.1058	Sel	No	No
LATS1	CRE.1279	Sel	No	No
LATS2	CRE.1740	No	Chimpanzee higher	No
PSMA4	CRE.438	Sel	No	No
PSMD11	CRE.1833	No	Human higher	No
PSMD14	CRE.735	No	Human higher	No
PSMD14	CRE.733	Sel	No	Human CRE higher in Neuron
PSMD14	CRE.734	Sel	No	No
STK4	CRE.1941	No	Chimpanzee higher	Human CRE higher in Neuron
STK4	CRE.834	Sel	No	Human CRE higher in Neuron
TLE3	CRE.433	No	Human higher	Human CRE higher in NPC
TLE3	CRE.434	Sel	No	No
TLE4	CRE.1506	No	Chimpanzee higher	No
WNT2	CRE.1350	Sel	No	No
WNT5A	CRE.893	Sel	No	No
WNT8B	CRE.118	Sel	No	No

Some genes in the Wnt signaling pathway were associated with multiple CREs or paralogous genes were associated with different CREs. CREs with signs of selection are indicated (sel), differential expression between orthologs (human or chimpanzee higher), and differential expression between cell types (higher in NPC or neuron).

Although there are only a relatively small number of core proteins involved in Wnt signal transduction, there are many proteins that help modulate signaling. In general, this happens through affecting Wnt binding to receptors, direct or indirect inhibition of *GSK3β*, control of *β-catenin* translocation, control of *β-catenin* association with the transcription activation complex, or inhibition of the transcription activation complex itself. CREs under selection and DE CREs are associated with genes involved in all of these processes (fig. 6). However, DE CREs are not enriched in any one part of the pathway and changes in CRE activity in humans do not tend to increase or decrease Wnt signal transduction. Interestingly, the only part of the core Wnt signaling pathway that has evidence of differential gene regulation is the *FZD1* receptor that helps to initiate Wnt signaling. Although we have only tested ∼20 of these CREs, and there are likely many regulatory regions that differ in activity around these genes, it does appear that DE CREs, with the exception of a DE CRE near *FZD1*, generally are associated with genes that act peripherally to the core pathway. Because Wnt signaling is critical in animal development, and modifications of the system could be deleterious, it is not surprising that we do not see much differential gene regulation in core components of the pathway. Yet, at the same time, this underscores the significance of adaptive changes occurring at the critical first step of Wnt signaling. Selection, however, does appear to be acting in regulatory regions throughout the Wnt pathway. CREs with signs of positive selection could be helping to control Wnt signaling in specific contexts by fine-tuning expression patterns. In comparison with selection in regulatory regions, protein coding regions of Wnt signaling genes are highly conserved. To test for signs of selection in coding regions, we calculated rates of nonsynonymous (d*N*) to synonymous (d*S*) substitutions in human sequences compared with chimpanzee for 31 of 47 genes for which Ensembl release 98 (Zerbino et al. 2018) data were available, and only found a d*N*/d*S* > 1 in *MYC*, which is a downstream effector of Wnt signaling.

Some genes in the Wnt signaling pathway were associated with multiple CREs or paralogous genes were associated with different CREs. CREs with signs of selection are indicated (sel), differential expression between orthologs (human or chimpanzee higher), and differential expression between cell types (higher in NPC or neuron).

Because we tested CREs in both NPCs and neurons, we were curious if we could see differences in the activity of CREs between cell types in the Wnt pathway. We tested human and chimpanzee CREs separately for differential activity in NPCs and neurons and found several that overlap with Wnt pathway genes. Most of these have higher activity in NPC with the exception of CREs associated with genes that compose the proteasome and STK4 that have higher activity in neurons (fig. 6). This could suggest that there is a higher level of regulation of Wnt signaling in less mature neural cells. The CREs that are more active in neurons are also less Wnt specific. STK4 is a kinase that is part of the MAP kinase signaling pathway, and the proteasome is a ubiquitous protein complex essential to cellular processes.

To look at how selection is working in Wnt pathway CREs, we aligned sequences between human and chimpanzee and looked for mutations that overlap with TF motifs from our earlier scan for JASPAR motifs. In many cases, mutations result in the appearance of new motifs in human CREs, but most often mutations modify existing motifs; we found 698 new motifs in 59 Wnt pathway CREs, and 1,572 existing motifs affected by mutations (supplementary table S5, Supplementary Material online). Among the motifs with new occurrences in human CREs, ZNF384 has the most new motifs, followed by Zfx, SP3, E2F4, TP73, IRF1, ZNF263, and TFAP2C(var.2), all with six or more new occurrences. The existing motifs that most frequently overlap mutations are ZNF384, ZNF263, EWSR1-FLI1, IRF1, SP2, and SP1, each with 24 or more instances affected by mutations. Interestingly, most of the existing and new motif occurrences are also some of the most frequently occurring motifs among all the CREs in our assay. These data suggest that there is not one specific subset of transcription factors that is driving evolution in regulatory elements around the Wnt pathway. Rather, changes in these CREs seem to be happening through modulation of existing binding sites with the appearance of additional sites that are already common in the genome.

## Discussion

Humans have experienced rapid evolution of neural phenotypes; yet, uncovering the adaptive genomic changes that control the developmental and functional processes responsible for these phenotypes has been one of the major challenges in evolutionary biology. Comparative genomics offers the opportunity to study evolution in the genome by comparing sequences between evolutionarily related species. Human–chimpanzee comparisons show a high similarity in coding sequences, suggesting that changes in gene regulation are responsible for phenotypic differences between species (King and Wilson 1975). Although the ability to identify putative regulatory elements in the genome has accelerated due to advances in high-throughput sequencing, understanding the physiological roles of these sequences remains an exciting challenge, and there is a growing field testing CREs in multiple species, cell types, and differentiation stages of neural iPCS (Doan et al. 2016; Ryu et al. 2018; Uebbing et al. 2021).

In this study, we combine computational and experimental genomic techniques to characterize the evolution and function of human neural enhancers in iPSC-derived NPCs and neurons that provide a physiologically relevant context for testing enhancers. By choosing highly CNSs that have accelerated evolution in humans (Pollard et al. 2006; Prabhakar et al. 2006; Lindblad-Toh et al. 2011) along with experimentally defined enhancers active in the developing or adult human brain (Vermunt et al. 2014; Reilly et al. 2015; Vermunt et al. 2016), we focused this assay on sequences that are enriched for neural and developmental processes which may be adaptive. We designed our assay with a global approach to include a broad set of enhancers that have putative functions in development or adult neural gene expression. In this way, we can explore how different subsets of enhancers have evolved to control neural phenotypes.

In our functional characterization, we identified 179 CREs that have significantly different activity between human and chimpanzee orthologs, and also found a set of 722 CREs under positive selection. Surprisingly, CREs in these two sets are largely nonoverlapping; only 34 are DE and have signs of positive selection. Furthermore, these CREs differ in genomic characteristics. Selection CREs are more distal to genes and other enhancers, are shorter sequences but belong to larger regulatory domains, and have lower GC content. When we looked at how TF motifs differ between humans and chimpanzees, we saw that selection CREs generally show more TF gains than losses, whereas motif gains and losses in human DE CREs are more balanced. The relationship between selection and DE is clearly complicated, with selection fine-tuning expression. There is some evidence of compensatory evolution in HARs and that individual variants in HARs increase or decrease activity may work to tune enhancers (Whalen et al. 2022). We also find that 40% of the HARs tested show signs of selection using our test. This is lower than other HAR studies, where selection was observed in ∼76% of HARs (reviewed in Levchenko et al. 2018). The differences in percentages may be due to using different types of tests for selection and different genome builds and outgroups used (Kostka et al. 2012).

In comparison with other MPRA studies looking at the evolution of CRE expression, we find some similar GO categories, such as development and cell differentiation (e.g. Uebbing et al. 2021; Whalen et al. 2022). In contrast, we also see categories related to adhesion, metabolic processes, and neural differentiation (fig. 2). This difference may be due to the different cell types analyzed, with the other studies using only neural precusor cells and here use differentiated neurons.

Our analysis of the genomic and TF motif characteristics of selection and DE CREs suggest functional differences between these CRE types, which agree with the patterns of expression that we observe in our assay. Selection CREs, which contain highly evolutionarily conserved sequences, have lower GC content than genomic background, which is associated with CNSs common among all mammals and is related to the composition of TF motifs within these sequences (Babarinde and Saitou 2013; Hettiarachchi and Saitou 2016). The preservation of ancestral sequences in selection CREs agrees with the observation that human accelerated enhancers have roles in regulating gene expression during development (Capra et al. 2013). Furthermore, the relatively short sequences that compose selection CREs belong to larger enhancer regions. This observation is in line with the model proposed by Emera et al. (2016) who show that highly CNSs shared among mammals are parts of larger enhancer regions, which contain both ancestral enhancer cores and adjacent linage-specific regulatory sequences. Together with the observation that selection CREs experienced more TF gains than losses in human sequences, this suggests that adaptive changes in selection CREs preserve ancestral functions that are critical for their roles in development. Human-specific changes in these sequences could provide ways to fine-tune activity without producing large changes in human sequences, which corresponds to our observation that there are few significant differences in activity between human and chimpanzee selection CREs (Whalen et al. 2022). These characteristics contrast with DE CREs that do not have highly conserved sequences, have GC content similar to background, and a higher rate of TF motif gains and losses. These data suggest that evolution of sequences in DE CREs is less constrained and that DE CREs are likely more recently evolved enhancers that could serve as a way to further define the level, location, or temporal dynamics of gene expression.

To look at how human CREs could be impacting neural phenotypes, we looked for genetic loci that contain CREs in our assay and GWAS SNPs associated with neuropsychiatric diseases. GWAS SNPs are often located in noncoding regions of the genome and have been shown to impact transcription factor motifs, linking these variants to putatively functional regulatory sites (Schaub et al. 2012; Gallagher and Chen-Plotkin 2018; Huo et al. 2019). Many of the CREs that we found in these loci are DE between humans and chimpanzee sequences. Although individual DE CREs do not fully explain the regulatory landscape in these loci, the overall patterns show that human gene regulatory elements evolved gains and losses of activity around neural genes. Understanding functional consequences of regulatory elements requires linking CREs to putative target genes, which is a challenge because enhancers can act distally and often have more than one target gene (ENCODE Project Consortium 2012). However, using multiple -omics tools and layering together these data sets can help us understand the larger context of regulatory function. First, many SNP variants associated with neuropsychiatric disease fall in noncoding regions, which implies that these loci function to regulate neural phenotypes. Second, the CREs in these loci are active brain enhancers, which connect these sequences to the regulation of neural processes. Third, these SNPs and CREs are contained within bioinformatically assigned gene regulatory domains, and these genes are enriched for neural and developmental processes. Fourth, Hi-C data show that CREs, SNPs, and nearby genes fall within TADs, and there is evidence of physical interaction between regulatory regions containing SNPs and CREs and gene promoters. These multiple lines of evidence underscore the functional roles of these loci in directing development and function of neural cells and suggest that many of the physiological processes affected by disease variants were also affected by evolutionary changes in the human genome. This also points to potential mechanisms for increased susceptibility to diseases that are much more common in humans; many of the evolutionarily changes that confer humans with unique phenotypes also increase the risk for diseases that disproportionately affect humans (Crespi 2010). Many of the neuropsychiatric diseases that are unique to humans may be the unavoidable consequence of variation in complex human traits (Stearns and Medzhitov 2016).

As we explored CREs associated with neural development and function, we found an abundance of CREs around genes associated with canonical Wnt signaling. Throughout the pathway, we found CREs with signs of positive selection and in general, DE CREs associated with genes that modulate Wnt signaling. One notable exception was a DE CRE with higher activity in the human ortholog associated with FZD1, which has been shown to be important for neurogenesis in the hippocampus (Mardones et al. 2016). FZD1 is DE in a single-cell study of iPSC-derived brain cell type humans and chimpanzees, but mainly in astrocytes and early in development (Kanton et al. 2019). Frizzled receptors are one of the primary receptors for Wnt ligands and mediate the initial events in the Wnt signaling cascade (MacDonald et al. 2009; Nusse and Clevers 2017). Work by Boyd et al. (2015) showed that a human accelerated enhancer, HARE5, upstream of FZD8, accelerates the cell cycle in neural progenitors leading to an increased number of neurons and greater cortical expansion in transgenic mice than the orthologous chimpanzee enhancer. In our assay, we also found CREs with signs of positive selection around three other frizzled paralogs, FZD5, 6, and 7. Because Wnt interaction with frizzled receptors initiates signaling, changes in these upstream signaling elements have potentially larger impacts on the signaling cascade that changes in downstream signaling elements, and the differential enhancer activity of HARE5 highlights the significant impacts that changes in frizzled receptors have on morphology of the developing neocortex. In our assay, we also found some differences in the *trans*-environment in which Wnt pathway CREs are active. We found more CREs with higher activity in NPCs than neurons, suggesting greater control of Wnt regulation in less mature neural cells. This differential regulation in neural progenitors corresponds with the role of Wnt signaling in controlling cellular proliferation. Together with the evidence for positive selection and differential CRE expression throughout the Wnt pathway, these data underscore the significance of the impacts that gene regulatory changes can have on driving differential neural phenotypes.

MPRAs are powerful tools for functionally testing large numbers of putative regulatory elements in a single assay (e.g. Doan et al. 2016; Ryu et al. 2018; Uebbing et al. 2021), and new approaches could examine larger chromatin regions (Girskis et al. 2021). Here we have applied it broadly to putative regulatory elements throughout the genome to examine the function of human neural enhancers. We have identified enhancers that have significant differences in activity between humans and chimpanzees that are strong candidates for follow-up validation with traditional reporter assays. Linking CREs to target genes with assays like chromatin conformation capture can further elucidate regulatory interactions and can be used to identify other enhancers that contribute to a gene’s regulatory network. The activity defined here, however, is only in one physiological context so a better understanding of when and where enhancers are active requires testing in other cell types or with in vivo assays. Understanding the larger genomic and cellular context of these functional enhancers will be important, elucidating the different roles they have played in human brain evolution and in neural disease susceptibility.

## Materials and Methods

### Library Design

We sought to assay a diverse set of putative regulatory elements that either show signs of positive selection in humans, are active during neural development, have higher activity in the human cortex than other regions, or have higher levels of active chromatin marks in the human brain compared with other primates. We chose noncoding regions with accelerated rates of nucleotide substitution in humans that were previously described by Pollard et al. (2006), Lindblad-Toh et al. (2011), Prabhakar et al. (2006), and Haygood et al. (2007), the “accelerated CREs,” and brain regions described by Reilly et al. (2015), and Vermunt et al. (2014, 2016) the “brain CREs.” Coordinates were converted to hg38 space using liftOver (Hinrichs et al. 2006) and sites in the accelerated or brain sets were merged to combine overlapping sequences. Sites in the brain set were filtered by size to include only those between 200 and 5,000 bp in length. To enrich our set for more distal regulatory elements, we removed sites from the brain set that were within 1 kb of Ensembl release 98 annotated TSSs (Zerbino et al. 2018). Any coordinates that were part of the accelerated set were removed from the brain set.

We used 171 bp sequences to construct our MPRA library. Regions smaller than this were appended with adjacent genomic regions, and 10 bp overlapping tiles were designed to cover larger regions. We first constructed tiles using hg38 coordinates and then mapped those to the chimpanzee (panTro5) genome using liftOver (Hinrichs et al. 2006). Human coordinates that did not map to chimpanzee were removed. To build oligos of the same length, chimpanzee sequences were designed to end 171 bp away from the liftOver-generated starting coordinate. We chose 1,579 accelerated sites covered by 2,857 oligos, and randomly chose 695 brain sites covered by 5,475 oligos. In total, the 100,000 sequences in the library represent 2,274 orthologous enhancers. To cover those sequences, we used 8,332 oligos per species × 2 species = 16,664 unique oligos to cover enhancers in both species. We used 6 barcodes per enhancer which, with negative controls, adds up to 100,000. Accelerated CREs were numbered CRE.1 through CRE.1579, and brain CREs were numbered CRE.1580 through CRE.2274 (supplementary table S7 and S10, Supplementary Material online).

Each 171 bp oligo sequence was represented in the library with six unique 11 bp barcodes. Sequences or barcodes containing ClaI or SalI restriction sites were removed. Barcodes were filtered to remove homotrimers, and GC content was either 45% or 55%. One nonsense control was included in the library and was represented with 16 unique barcodes. The library was thus composed of 100,000 sequences. CRE and barcode sequences were separated by an 18 bp spacer sequence including ClaI and SalI restriction sites that were used to add a minimal promoter and EGFP reporter, and thereby position the CRE sequences upstream of the promoter and barcodes in the EGFP 3′-UTR. The 15 bp primer binding sites (Inoue et al. 2017) were added at the ends of the oligos to yield 230 bp oligo sequences.

### Cloning Oligo Library into Lentiviral Vector

A lentiviral reporter vector backbone was constructed from the pGPG lentiviral transfer plasmid provided by the UMass Worcester Viral Vector Core. The CMV promoter within pGPG was removed by ClaI and XbaI digestion. A minimal promoter from pGL4.23 was PCR amplified with primers adding ClaI and XbaI restriction sites. This minimal promoter was ClaI and XbaI digested and cloned into pGPG to produce the reporter backbone pGPG_mP. The vector was digested with ClaI and SalI, and the minimal promoter-EGFP fragment and the linear pGPG backbone were gel purified.

Array synthesized oligos were run on a 10% Tris-borate-EDTA-urea denaturing polyacrylamide gel, stained with SYBR Gold, and 230-mer bands were excised and resuspended in TE buffer. Gel-purified oligos were amplified with emulsion PCR to add vector-complementary tails that abolish ClaI and SalI sites. Oligos were inserted into the linear pGPG backbone with Gibson assembly to produce the library pCRE. The pCRE library was ClaI and SalI digested and the previously excised minimal promoter-EGFP fragment was reintroduced by sticky end ligation to produce library pMPRA. Both libraries, pCRE and pMPRA, were sequenced on an Illumina MiSeq to assess oligo quality and library diversity.

### Library Sequencing

Library pCRE was sequenced on an Illumina MiSeq using 2 × 250 bp paired-end reads to assess library quality. Illumina adapter sequences and custom read primers (supplementary table S6, Supplementary Material online) were added to CRE-barcode sequences in the pCRE library using emulsion PCR with primers pCRE_adpt_F/R to bind 15 bp sites at the ends of the designed oligos (supplementary table S6, Supplementary Material online). A total of 8.5 million paired-end reads were generated. About 91% of the designed oligos were present in the pCRE library and 72% of designed oligos have at least one perfect match.

Library pMPRA was sequenced on an Illumina MiSeq using 300 bp paired-end reads to assess oligo abundance in the final library. CRE regions were amplified with primer pMPRA_QC_F/R (supplementary table S6, Supplementary Material online) that binds opposite sides of the CRE insert. Amplicons were prepared for Illumina sequencing with NEBNext Ultra II DNA Library Prep kit (NEB, E7645) and sequenced using standard Illumina primers. A total of 31 million reads were generated for 12,777 unique oligos, representing ∼77% of the designed library. All fastq files are uploaded and embargoed on the NCBI short read archive (BioProject PRJNA638914). The link for reviewers is:

https://dataview.ncbi.nlm.nih.gov/object/PRJNA638914?reviewer=s9ehfuf8klns1eq9atginb0qbc

### Cell Culture and Transduction

Neural progenitor cells and neurons were differentiated from iPSCs that were from the Gilad lab at the University of Chicago. In total, six iPSC lines, three human and three chimpanzee (supplementary table S7, Supplementary Material online), were used to produce NPCs and neurons. iPSCs were initially cultured in mTeSR1 medium (STEMCELL Technologies), and were subsequently cultured in neural induction medium for ∼3 weeks to produce NPCs. Neurons were differentiated from NPCs by culturing for 1 week in neural differentiation medium (STEMCELL Technologies), and 1 week in neural maturation medium (STEMCELL Technologies). NPCs and neurons of each cell line were cultured in triplicate in six-well plates until cells were ∼90% confluent. Library pMPRA, packaged into lentiviral particles by the UMass Worcester Viral Vector Core, was used to transduce cells at a multiplicity of infection of 10 by replacing medium for medium containing virus. Medium was removed and replaced 24 h after infection. Cells were washed, trypsinized, and collected by centrifugation 48 h after infection.

### RNA and DNA Library Preparation and Sequencing

Total RNA and DNA were collected from each replicate with a Qiagen AllPrep kit (Cat 80204). NPC cell pellets were lysed by vortexing in buffer RLT with β-mercaptoethanol. Neuron cell pellets were lysed in a TissueLyser II at 30 Hz for 1 min in buffer RLT with β-mercaptoethanol. cDNA was synthesized from 1,500 ng of total RNA from each replicate with SuperScript III (Invitrogen) using random primers. PCR to amplify barcodes from DNA and cDNA was carried out using NEB Phusion polymerase and primers MPRA_seq_F&R (supplementary table S6, Supplementary Material online): 98 °C for 30 s, 25 cycles of (98 °C for 10 s, 63 °C for 30 s, and 72 °C for 10 s), and 72 °C for 5 min. Two 25 μL reactions were performed for each replicate and PCR amplification of barcodes was performed separately for each set of replicates from each cell line to avoid cross-contamination between samples. Following amplification, replicates were pooled, size-selected, and purified with AmpureXP beads (Beckman-Coulter). Libraries for Illumina sequencing were prepared with 250 ng of barcode amplicons using NEBNext Ultra II DNA Library Prep kit (NEB, E7645). Sequencing was performed on an Illumina NextSeq to generate 2 × 150 bp overlapping paired-end reads. Libraries from NPCs and neurons were sequenced in separate assays. To obtain higher read coverage in cDNA libraries, 50% more cDNA than DNA was added to the pool. In total, we obtained 67M reads for NPCs and 77M reads for neurons.

### Assessing CRE Activity

To obtain counts for RNA and DNA from each CRE, we began by merging forward and reverse read pairs with NGmerge (Gaspar 2018). We counted barcodes from reads if the barcode sequence and three adjacent base pairs on both sides matched the designed sequence exactly. We further filtered reads to include only those with *q*-values 14 or greater (*P* < 0.05) for every base in the barcode. Within each replicate, we counted the occurrence of each oligo from the RNA and DNA reads. We only kept RNA reads if DNA for the same oligo was sequenced in the same replicate. The sum of RNA and DNA counts was calculated for all oligos tiled across each CRE for each replicate. To account for different sequencing depth between replicates, we calculated counts per million for RNA and DNA counts and then calculated the ratio of RNA to DNA for each CRE (supplementary table S8, Supplementary Material online). There were 1,157 orthologous CRE pairs tested in the assay of the 2,274 in the design (supplementary fig. S5, Supplementary Material online). We counted CREs as “active” if they were expressed in at least two replicates of either species cell type. About 268 CREs passed this stringent filter and were used for differential expression analysis. To assess activity between orthologs, we used limma (Ritchie et al. 2015) to test for significant differences in expression across all 12 biological replicates for human and chimpanzee CREs and found 179 DE CREs; 100 with higher expression from the human ortholog, and 79 with higher expression from the chimpanzee ortholog. Differences in expression between neurons and NPCs were tested separately for human CREs and chimpanzee CREs by comparing expression across all six biological replicates of each cell type.

### Selection Testing

We tested CREs for signs of putative positive selection using a modified version of the method described by Haygood et al. (2007). This test uses CRE sequence alignments (or any noncoding sequence) to determine nucleotide substitution rates in a specific sequence and then uses another sequence alignment of both intergenic and intronic regions within 100 kb of the test sequence and determines a neutral substitution rate, with 50 kb on each side.

A low *P*-value requires that some but not all or even most promoter sites have evolved more quickly than the average intronic site and that the null model accommodates putative promoter sites that have evolved under negative selection on the chimpanzee and macaque lineages but neutrally on the human lineage. Thus, the contrast between the models is sensitive to positive selection rather than mere relaxation of negative selection. If the substitution rate in the CRE sequence is higher than in the neutral sequence, positive selection is inferred. The code to execute this script is available on GitHub (https://github.com/jpizzollo/TestForPositiveSelection) and is executable using HyPhy (Pond et al. 2005).

### CRE-Gene Assignments and GO Analysis

In order to explore putative functions of CREs, we assigned CREs to genes using GREAT (McLean et al. 2010) with the “basal plus extension” method, including curated regulatory domains. This method defines regulatory domains for genes and assigns these genes and their annotations to CREs that fall within the defined regulatory domains. For selected sets of CREs (all, tested, active, DE, and selection CREs), we used the GREAT-associated genes to perform functional enrichment analysis for GO biological processes using gProfiler (Raudvere et al. 2019). We used gProfiler’s g:SCS algorithm that adjusts *P*-values for multiple testing to assign a significance threshold (*q*-value) of 0.05 to category enrichments. Enriched GO terms were grouped into similar parent categories (e.g., cell–cell signaling, adhesion, regulation of transcription, etc.) based on term names (fig. 2*B*). Semantic similarity measures were calculated with GOSemSim (Yu et al. 2010) using the enriched Term IDs output from gProfiler.

### Assessing Genomic Distribution of CREs

To compare the gene density in proximity to CREs, we counted TSSs within 1 Mb of either the 5′- or 3′-end of CREs. Ensembl-annotated TSS data were obtained from the GRCh38 data set in Biomart (Zerbino et al. 2018). To assess the number of putative enhancers within 1 Mb of either the 5′- or 3′-end of CREs, we used H3K27ac and H3K4me2 ChIP-seq data human embryonic cortex (Reilly et al. 2015) and H3K27ac ChIP-seq data from adult human brain (Vermunt et al. 2014). From the Reilly et al. (2015) data set, we only chose sequences that had both acetylation and methylation marks in the same replicate at any time point. From the Vermunt et al. (2014) data set, we chose sequences with acetylation marks in at least two biological replicates from any of the brain regions tested. To compare the size of the regulatory domains that contain the CREs in our assay, we used the same Reilly et al. (2015) and Vermunt et al. (2014) data sets and used the bedtools intersect command to look for overlap between CREs in our assay and functional regulatory domains.

### Assessing GC Content and CpG Motif Abundance

We calculated the GC content and CpG motif abundance in all CREs in our assay by directly analyzing the sequences of our CREs. We obtained CRE sequences from the hg38 genome downloaded from the UCSC table browser (Karolchik et al. 2004) and directly counted the occurrences of C and G nucleotides and CpG dinucleotides to determine their abundance.

### TF Motif Identification and Comparison between Species

To look for TF motifs, we scanned human and chimpanzee CREs for the presence of 579 experimentally defined transcription factor binding sites described in the JASPAR CORE Vertebrate database (Khan et al. 2018). We looked for these motifs with the FIMO package from the MEME suite (Grant et al. 2011) using sequences for all human or chimpanzee CREs as background when scanning for motifs in either species, and used a *P*-value threshold of 10^−4^. To determine if TF motifs are more enriched in CREs than other noncoding regions, we scanned a random set of 1,000 human and chimpanzee intergenic or intronic sequences for JASPAR motifs using the same parameters as our scan in CRE sequences. Random sequences were chosen by collecting all intergenic and intronic regions, excluding first introns, removing sequences <500 bp, and taking only the first 5,000 bp of longer sequences.

To look at how motifs are changing between species, we counted the total number of occurrences of each motif for each species within each CRE set and then calculated the fold change in motif frequency between species. This comparison of motif frequency across all background CREs provides the global frequency change between species. This same comparison in selection or DE CREs, however, will also show motif differences that are due to global motif frequency differences between species. To account for this, we calculated the difference in between-species motif fold change between selection or DE CREs and background CREs. Then, to identify the motifs that have more changes between species in CRE subsets than background, we chose motifs for which the difference between selection or DE CREs and background CREs was >2 standard deviations from the mean difference.

### Correlating Gene Expression and CRE Activity

Total RNA was extracted from cells using an RNeasy Plus Mini kit (Qiagen), including a DNase step to remove residual DNA. Total RNA was analyzed for quality using the Agilent Bioanalyzer system (Agilent RNA 6000 Nano kit) with RNA integrity numbers for all samples between 8.3 and 10 (supplementary table S1, Supplementary Material online). Using the NEBNext Poly(A) magnetic mRNA isolation kit (NEB), mRNA was isolated from intact total RNA, and cDNA libraries were made from each sample using the NEBNext RNA Ultra II Library Prep kit for Illumina (New England Biolabs). Barcoded samples were sequenced using the Illumina NextSeq 500 platform at the Genomics Resource Core Facility (Institute for Applied Life Sciences, UMass Amherst) to produce 75 bp single-end reads, yielding a minimum of 32 million reads per sample. The activity of each gene was averaged from raw counts across each of three species-cell-type replicates (e.g., three human NPCs) as an overall measure of gene expression. The activity of each CRE was averaged from the RNA/DNA ratios across all six cell-type replicates for each species-oligo (e.g., human CREs tested in three human NPCs and three chimpanzee NPCs). An overall correlation between CRE activity and gene expression was tested using a Spearman’s test, combining activity of all human CREs and all chimpanzee CREs compared with human gene expression and chimpanzee gene expression, respectively.

### Associating CREs with GWAS SNPs and Visualizing Chromatin Contacts

SNPs associated with neuropsychiatric diseases were obtained from the NHGRI-EBI GWAS catalog (Buniello et al. 2019) by searching disease terms for Alzheimer’s disease, autism, attention deficit hyperactivity disorder, bipolar disorder, major depressive disorder, or schizophrenia. To identify SNPs and CREs that occur in the same loci, we assigned SNPs and CREs that were active in our assay to nearby genes using GREAT (McLean et al. 2010) with the “basal plus extension” method, including curated regulatory domains. We then intersected the genes associated with SNPs and CREs to find loci that contain GWAS variants and putative regulatory elements. To test for enrichment of SNPs among DE CREs, we compared the number of SNPs associated to DE or all CREs in our assay that mapped to genes using GREAT, and then performed a fisher exact test to generate a statistic for the SNP-CRE associations. To visualize genomic contacts within GWAS loci, we used Hi-C data (Schmitt et al. 2016) from human prefrontal cortex, which were downloaded from the 3D Genome Browser (Wang et al. 2018). The downloaded images included schematics of gene locations within these loci. These annotations were condensed to optimize space in the figure and unannotated transcripts were removed.

### Associating CREs with Genes in the Wnt Signaling Pathway

To explore the regulatory regions that could be impacting genes in the Wnt pathway, we first collected a set of genes that have Ensembl-defined orthologs in human and chimpanzee that are associated with canonical Wnt signaling. We included all genes that were part of the “Wnt signaling,” “GO canonical Wnt signaling pathway,” and “KEGG Wnt signaling pathway” gene sets in mSigDB (Subramanian et al. 2005). We then checked if any of the CREs in our assay were located within 500 kb of the TSSs of Wnt pathway genes.

## Supplementary Material

evac108_Supplementary_Data

## Data Availability

All fastq files are uploaded and embargoed on the NCBI short read archive SUB7553721: https://submit.ncbi.nlm.nih.gov/subs/sra/SUB7553721/overview
