# Supplementary material for: Differentially Active and Conserved Neural Enhancers Define Two Forms of Adaptive Noncoding Evolution in Humans
Source: Genome Biol Evol. 2022 Jul 22;14(8):evac108. doi: 10.1093/gbe/evac108 (PMC9348619; doi:10.1093/gbe/evac108)
Supplement: evac108_Supplementary_Data [file evac108_supplementary_data.zip › List of Supplemental Information.docx]

**List of Supplemental Tables:**

TS1. TF motifs with the most different frequencies between human and chimpanzee.

TS2. Table listing CREs to nearest genes and SNPs analyzed.

TS3. Gene, CRE, and if they are a selection CRE or a brain CRE, and DE.

TS4. Human motif gains in Wnt-pathway CREs

TS5. Primers used in MPRA construction.

TS6. Cell line information.

TS7. A bed file of all tested CRE locations in the human genome.

TS8. Differential expression of the CREs examined. P-values were used in the analysis, but the adjusted p-values (FDR 5%) are reported here.

TS9. CREs showing DE and if they are accelerated or brain.

TS10. Original studies CREs were identified in.

List of Supplemental Figures:

FS1. Gene enrichments for different categories and samples included in the study.

FS2. CRE Activity vs GC Content by Ortholog and CRE Set

FS3. CpG density by Set in humans and chimpanzees.

FS4. Tests of differences by set and TFBS motif density.

FS5. Percentage of accelerated vs brain CREs in each subset of CREs.
