# Supplementary figures and images for: Differentially Active and Conserved Neural Enhancers Define Two Forms of Adaptive Noncoding Evolution in Humans
Source: Genome Biol Evol. 2022 Jul 22;14(8):evac108. doi: 10.1093/gbe/evac108 (PMC9348619; doi:10.1093/gbe/evac108)

Supplementary Firure 1

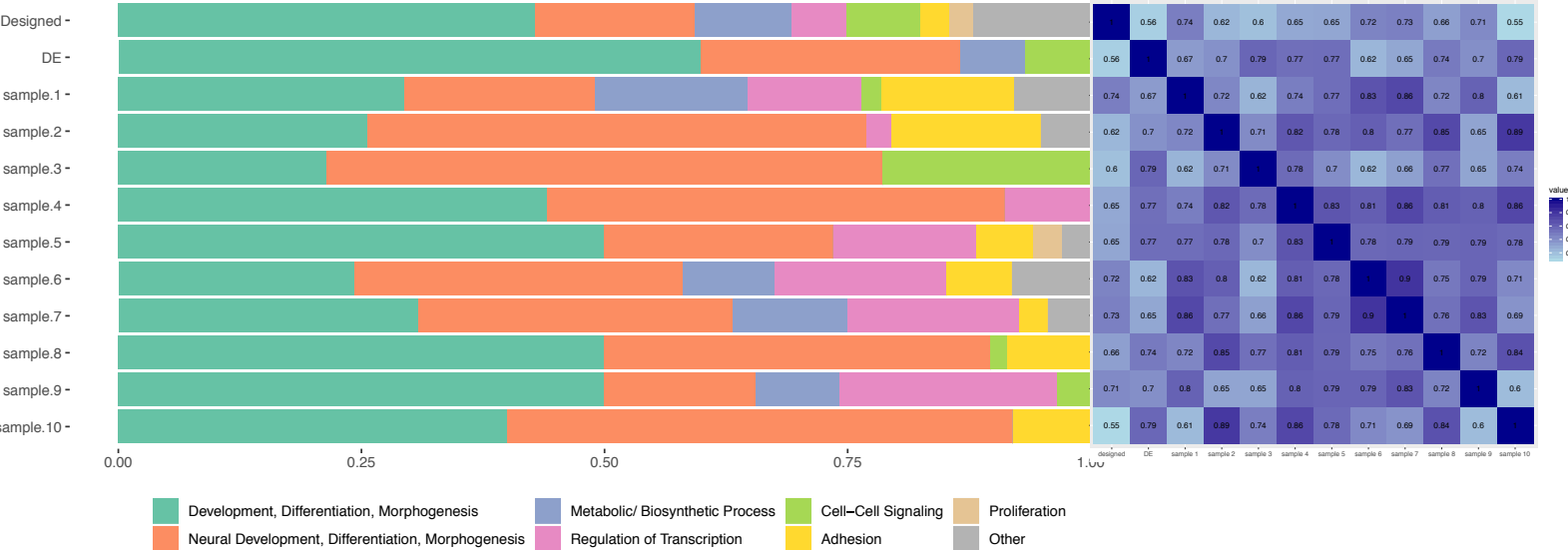

Supplement: evac108_Supplementary_Data [file evac108_supplementary_data.zip › SF1.pdf]

# Supplementary Figure 2

CRE Activity vs GC Content by Ortholog and CRE Set

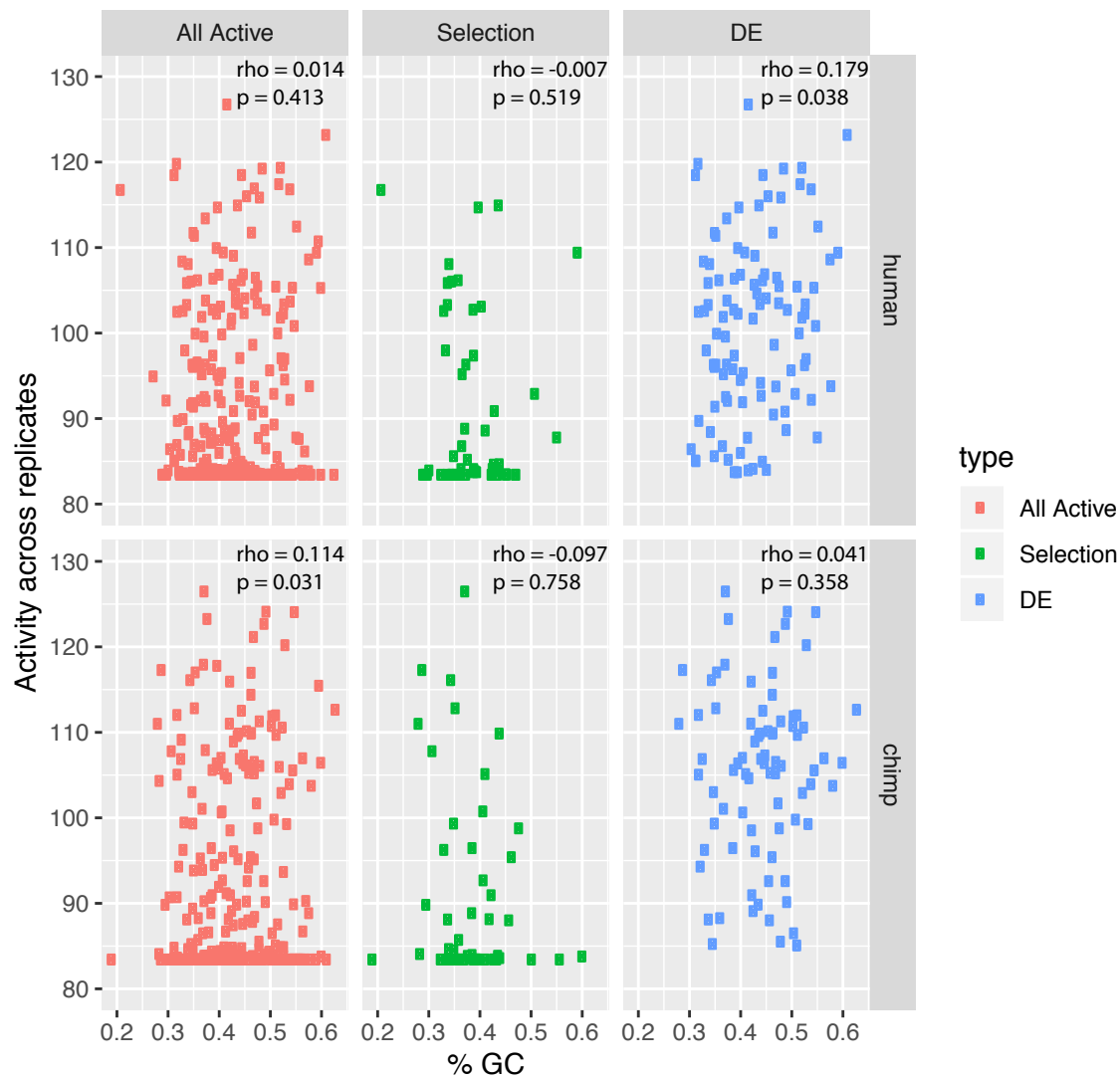

Supplement: evac108_Supplementary_Data [file evac108_supplementary_data.zip › SF2.pdf]

### Supplementary Figure 3

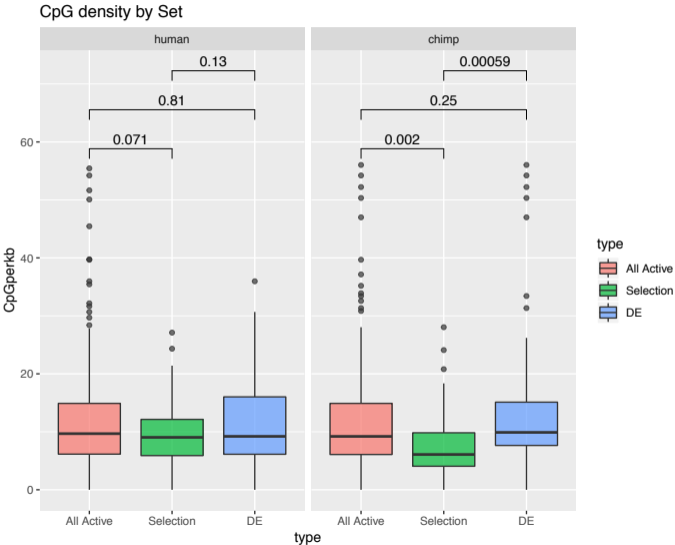

Supplement: evac108_Supplementary_Data [file evac108_supplementary_data.zip › SF3.pdf]

# Supplementary Figure 4

A

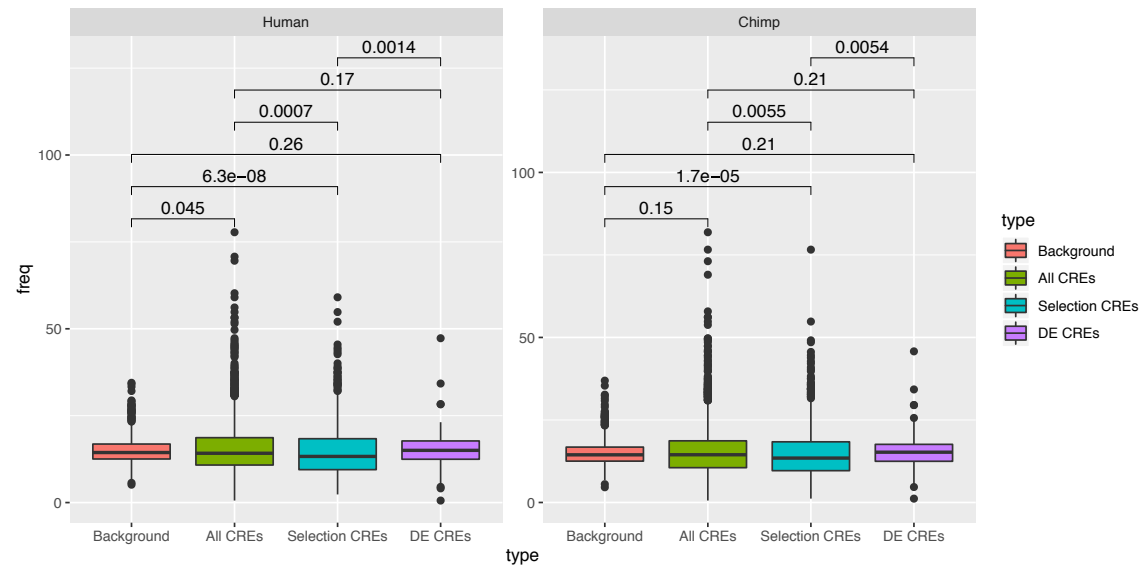

B

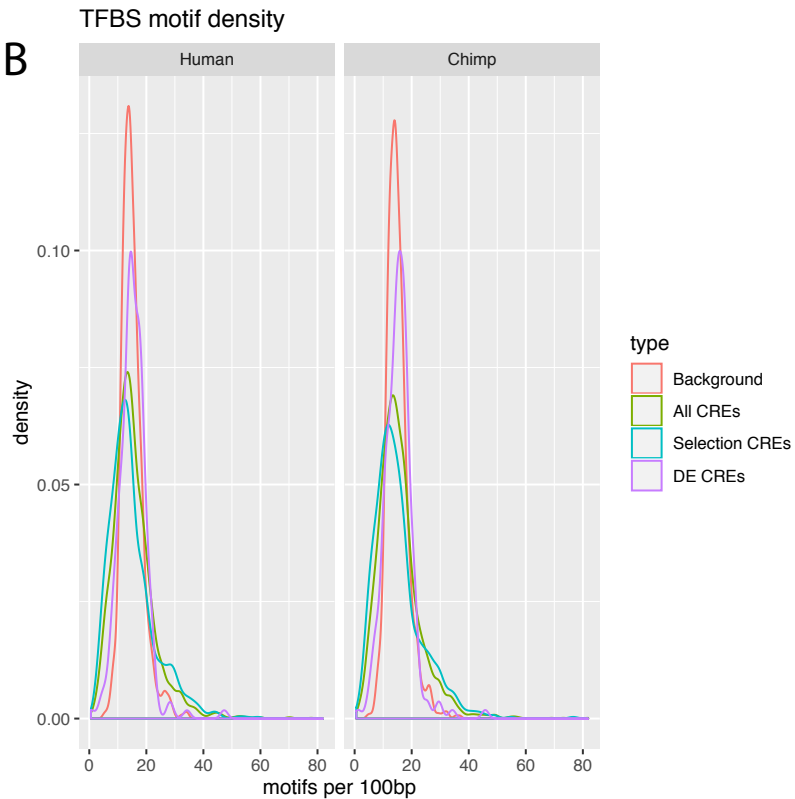

Supplement: evac108_Supplementary_Data [file evac108_supplementary_data.zip › SF4.pdf]

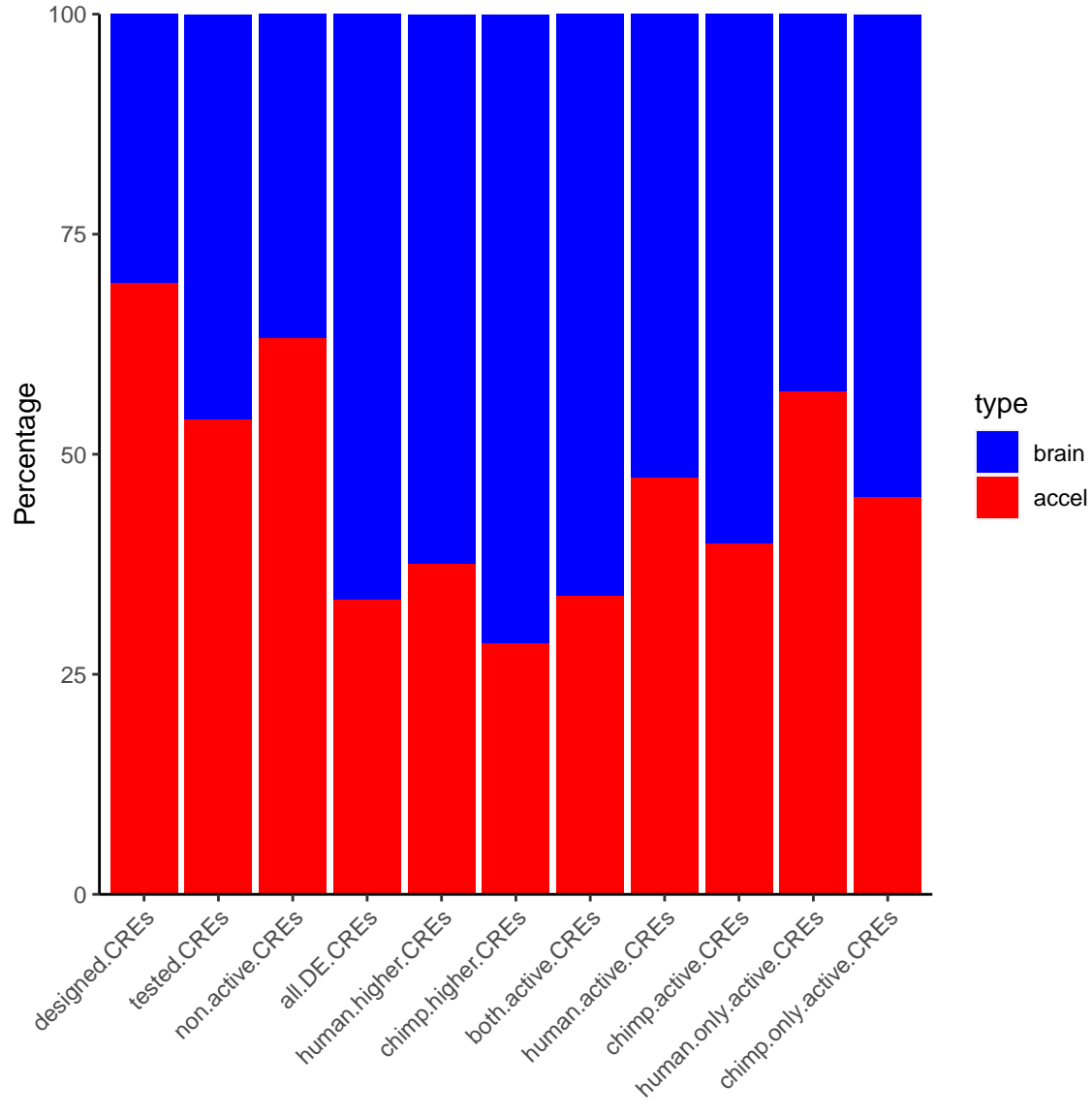

Supplement: evac108_Supplementary_Data [file evac108_supplementary_data.zip › SF5.pdf]
